# Renal Protection by Genetic Deletion of the Atypical Chemokine Receptor ACKR2 in Diabetic OVE Mice

**DOI:** 10.1155/2016/5362506

**Published:** 2015-12-20

**Authors:** Shirong Zheng, Susan Coventry, Lu Cai, David W. Powell, Venkatakrishna R. Jala, Bodduluri Haribabu, Paul N. Epstein

**Affiliations:** ^1^Department of Pediatrics, University of Louisville, Louisville, KY 40202, USA; ^2^Department of Pathology, University of Louisville, Louisville, KY 40202, USA; ^3^Department of Medicine, University of Louisville, Louisville, KY 40202, USA; ^4^Department of Microbiology and Immunology, University of Louisville, Louisville, KY 40202, USA

## Abstract

In diabetic nephropathy (DN) proinflammatory chemokines and leukocyte infiltration correlate with tubulointerstitial injury and declining renal function. The atypical chemokine receptor ACKR2 is a chemokine scavenger receptor which binds and sequesters many inflammatory CC chemokines but does not transduce typical G-protein mediated signaling events. ACKR2 is known to regulate diverse inflammatory diseases but its role in DN has not been tested. In this study, we utilized ACKR2^−/−^ mice to test whether ACKR2 elimination alters progression of diabetic kidney disease. Elimination of ACKR2 greatly reduced DN in OVE26 mice, an established DN model. Albuminuria was significantly lower at 2, 4, and 6 months of age. ACKR2 deletion did not affect diabetic blood glucose levels but significantly decreased parameters of renal inflammation including leukocyte infiltration and fibrosis. Activation of pathways that increase inflammatory gene expression was attenuated. Human biopsies stained with ACKR2 antibody revealed increased staining in diabetic kidney, especially in some tubule and interstitial cells. The results demonstrate a significant interaction between diabetes and ACKR2 protein in the kidney. Unexpectedly, ACKR2 deletion reduced renal inflammation in diabetes and the ultimate response was a high degree of protection from diabetic nephropathy.

## 1. Introduction

Although hyperglycemia is the initiating and essential cause for all diabetic complications there is accumulating evidence that inflammatory processes activated by chronic elevated glucose are integral to the development of diabetic complications [1]. Diabetic nephropathy (DN) is one of the most severe and common complications of diabetes and it is the leading cause of end stage renal failure in the world. Immune modulation and inflammatory process contribute to the development and progression of DN [2, 3]. In diabetic kidneys expression of proinflammatory chemokines rises and infiltration of inflammatory cells increases [4–7]. These changes are correlated with progression of tubulointerstitial injury and deterioration of kidney function [8–10]. Inhibition of renal inflammation by small molecule inhibitors or by antibodies directed against chemokines or chemokine receptors has been shown to reduce renal damage in DN [11–14]. More complete understanding of how the kidney modulates immune and inflammatory processes in diabetes may lead to the discovery of improved biomarkers and new therapeutic targets for treatment of DN.

ACKR2 is a chemokine decoy receptor [15] which can bind and internalize chemokines without activating an intracellular response [16]. ACKR2 binds most inflammatory CC-chemokines (CCL2, CCL5, CCL3, CCL4, CCL7, CCL8, CCL11, CCL13, CCL17, CCL22, CCL23, and CCL24) leading to their degradation, thereby reducing local levels of inflammatory chemokines. This makes ACKR2 a likely modulator of local inflammation. The function of ACKR2 has been tested in knockout animals in which deletion of ACKR2 coding sequences increased the inflammatory response in cutaneous tissue [17], placenta [18], lung [19], liver [20], and colon [21]. The role of ACKR2 has not been examined for a complication of diabetes. In this study, we examined the effect of crossing an established ACKR2 knockout mouse (designated herein as ACKR2 mice) with the diabetic mouse model, OVE26 (OVE). This diabetic model exhibits several features of human DN [22] and extensive renal inflammation [23, 24].

## 2. Methods

### 2.1. Animals

All animal procedures followed the NIH Guide for the Care and Use of Laboratory Animals and were approved by the University of Louisville Institutional Animal Care and Use Committee. ACKR2 mice on the C57BL/6 background originally from Charles River Italia (Calco, Italy) [17] were bred to FVB mice for at least 10 generations to transfer the ACKR2 deletion to the FVB background (henceforth designated as ACKR2). These ACKR2 mice were bred for two generations to diabetic OVE mice on the background FVB to produce OVE mice homozygous for the ACKR2 deletion (OVE-ACKR2). Mice were maintained up to 6 months of age. Animals had free access to standard rodent chow and water throughout the study.

### 2.2. Glucose and Albumin Assays

Glucose was assayed in serum samples obtained from nonfasted mice at 6 months of age by the Glucose (HK) Assay Kit (Sigma-Aldrich). At 2 months urine glucose was evaluated with Clinistix (Bayer). Albumin was measured from spot urine samples with a mouse albumin ELISA kit (Bethyl Laboratories, Montgomery, TX) within the linear range of the assay. Urine creatinine was measured with a creatinine assay kit (DICT-500, BioAssay Systems). Urine albumin was expressed as the ratio of albumin to creatinine (*μ*g/mg).

### 2.3. Assessment of Renal Fibrosis and Inflammatory Cell Infiltration

Kidneys were fixed overnight in 10% neutral buffered formalin and embedded in paraffin. Sagittal tissue sections from the center of the kidney were stained with Masson's trichrome using standard protocols. Stained slides were imaged with a 20x objective. Fibrosis was semiquantitatively scored by a blinded observer for the number of blue stained fibrotic areas per section. Renal inflammatory cell infiltration was evaluated by staining sections with rat anti-mouse CD45 antibody (Angio-Proteomie, Boston, MA). Positive staining was detected with HRP conjugated second antibody and diaminobenzidine (DAB). CD45 positive cell infiltration was evaluated by quantitating the DAB stained pixel area in 8 random, nonoverlapping 200x image fields from the cortical region per mouse with 3 mice per group. Digital images were taken by an observer blind to the identity of the section and the number of positive pixels was quantified by another observer blind to section identity. Pixel number was determined using the ability of Adobe Photoshop to select areas of matching color intensity.

### 2.4. Microarray Hybridization and Gene Expression Analysis

RNA extraction was done with the RNeasy Mini Kit (Qiagen, Santa Clarita, CA, USA) from frozen kidneys. Extracted RNA was checked for quality on Agilent 2100 Bioanalyzer (Agilent Technologies, Palo Alto, USA). The RNA samples having RNA integrity number (RIN) above 8.8 (average 9.1) were used for probe preparation. A 100 ng aliquot of RNA from each mouse was used for probe preparation with an Ambion WT Expression kit. The kit generates sense-strand cDNA from total RNA for fragmentation and labeling was done with an Affymetrix GeneChip WT Terminal Labeling Kit (PN90067). Probes from 3 six-month-old female mice in each group were hybridized to Affymetrix mouse gene 1.0 ST exon arrays and scanned with a GCS 3000 7G scanner and signals were analyzed with Command Console software (Affymetrix, Santa Clara, CA). Gene expression profiles were uploaded to Ingenuity software (Ingenuity Systems, http://www.ingenuity.com/, Ingenuity Pathway Analysis, Redwood City, CA) for data analysis. Gene array data was uploaded to GEO and the access number is GSE51205.

### 2.5. Quantitative Reverse Transcription-PCR

Total RNA was extracted from whole kidney using TRIzol reagent (Invitrogen, Carlsbad, CA). The cDNA was synthesized with high-capacity cDNA archive kit (p/n 4322171, Applied Biosystems, Foster City, CA) and PCR was performed on an Applied Biosystems 7300 thermocycler with commercially available Taqman reagents (Assay on Demand, Applied Biosystems) for ccbp2 (ACKR2) (Mm00445551_m1), ccl2 (Mm00441242_m1), ccl5 (Mm01302428_m1), ccr2 (Mm04207877_m1), and ccr5 (Mm01216171_m1). Amplification was performed in duplicate using 40 cycles of denaturation at 95°C for 15 sec and primer annealing/extension at 60°C for 1 min. Expression data were normalized to 18s ribosomal RNA (Hs99999901-sl) or GAPDH RNA measured on the same samples. Relative expression ratio was calculated according to the 2^−ΔΔCT^ method.

### 2.6. ACKR2 Immunohistochemistry Staining in Human Kidney

Immunohistochemistry with anti-human ACKR2 antibody was used for detection of ACKR2 expression in human kidneys: renal tissue biopsies (*n* = 9) from diabetic patients with confirmed diabetic nephropathy and 6 nondiabetic control renal tissue samples (2 donor kidneys, 1 normal portion from renal cancer patient, and 3 renal biopsy specimens with proteinuria, lacking visible tubulointerstitial alterations). The research protocol was approved by our Medical Ethics Committee. Tissue was embedded in paraffin, stained with rat anti-human ACKR2 antibody (R&D SYSTEMS, Inc., Minneapolis, MN), and detected with DAB. ACKR2 staining in each section was scored semiquantitatively in tubular and interstitial regions with the criteria of 0 for none, 1 for rare, 2 for some, 3 for common, and 4 for common plus intense. The scorer had no knowledge of group identification of the slides. ACKR2 expression was presented as the average score of each group. In some samples tissues were double labeled with FITC conjugated* Lotus tetragonolobus* lectin (Vector labs), a marker for tubule epithelial cell brush border [25], and the ACKR2 antibody binding was visualized with Cy3 conjugated anti-rat second antibody.

### 2.7. Statistical Analyses

Data are expressed as means ± SE. Comparisons between two groups were performed by *t*-test. Comparisons between more than 2 groups were performed by one-way ANOVA. Statistical analyses were performed with SigmaStat software.

## 3. Results

### 3.1. ACKR2 Deletion Did Not Alter Diabetes Development in OVE26 Mice

**Figure 1:**
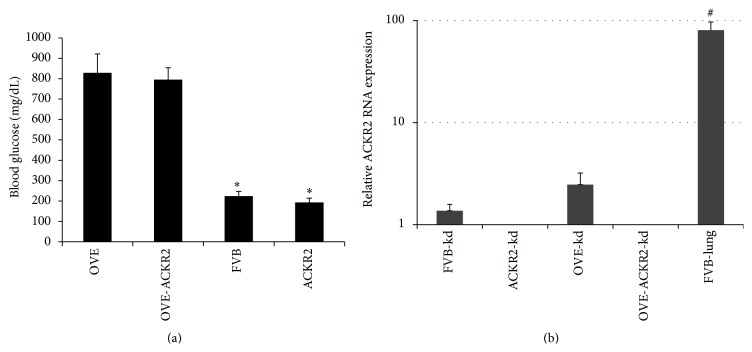
Blood glucose and ACKR2 RNA in diabetic and normal mice, with and without deletion of the ACKR2 gene. (a) ACKR2 knockout did not affect blood glucose levels in free fed normal or diabetic mice. ^*∗*^ *p* < 0.02 for both nondiabetic groups versus both diabetic groups. *N* = 4,6, 6, and 11 in FVB, ACKR2, OVE, and OVE-ACKR2 groups, respectively. (b) Low level ACKR2 RNA expression in kidney is eliminated in ACKR2 KO mice. No ACKR2 RNA was detected in any ACKR2 kidney sample. *N* = 4 for nondiabetic kidney groups, 3 for diabetic kidney groups, and 2 for normal lung. Data are presented on a log⁡10 graph to include expression values for lung. # indicates that ACKR2 RNA expression in FVB lung was significantly higher than in normal FVB kidney. ACKR2 expression in diabetic kidney tended to be higher than in FVB kidney.

Enzymatic assays, necessary for accurate measurement of blood glucose in OVE diabetic mice [22, 26], indicated that deletion of the ACKR2 gene did not significantly reduce blood glucose levels in 6-month-old OVE-ACKR2^−/−^ mice (Figure 1(a)). Urine glucose, undetectable in normal mice, exceeded 2000 mg/dL at 2 months of age in all OVE and OVE-ACKR2 spot urine samples tested (*n* = 4 per group, data not shown). Expression of ACKR2 RNA was 80% higher in diabetic kidneys compared to normal kidneys (Figure 1(b)), though this difference was not significant (*p* = 0.11). Interestingly high levels of ACKR2 in lungs were observed. Knockout mice served as negative controls for expression analysis.

#### 3.1.1. Knockout of the ACKR2 Gene Reduced Diabetic Albuminuria

**Figure 2:**
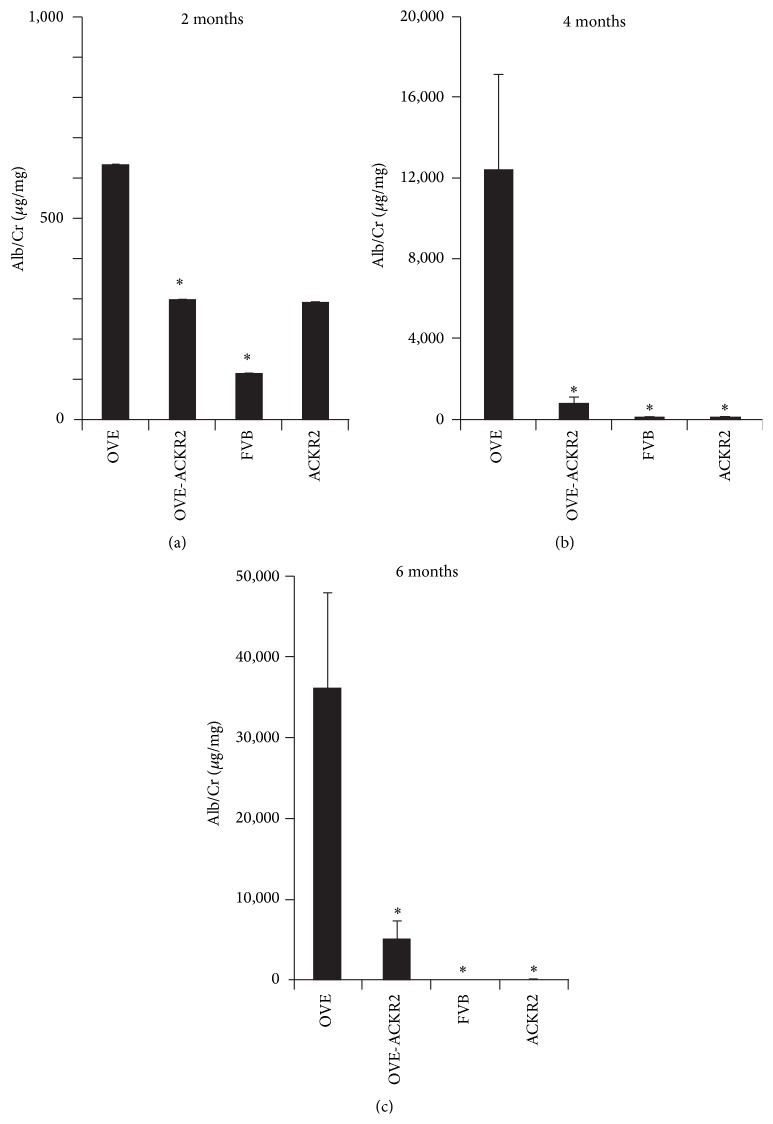
Diabetic albuminuria was reduced by knockout of the ACKR2 gene at 2, 4, and 6 months of age. Urine albumin and creatinine were determined as described in Methods. *∗* indicates *p* < 0.05 versus OVE. Comparisons were performed by one-way ANOVA. *n* ≥ 12 in each OVE and OVE-ACKR2 group. For FVB *n* = 14,9, and 6 at 2, 4, and 6 months, respectively. For ACKR2 *n* = 3, 7, and 7 at 2, 4, and 6 months, respectively.

Albuminuria was assessed by measuring albumin/creatinine ratio (ACR expressed as *μ*g/mg) in all groups at 2, 4, and 6 months of age (Figure 2). By 2 months ACR was already significantly elevated in OVE mice compared to FVB controls. ACR increased in OVE mice with age, from 600 at 2 months to over 10,000 at 4 months and over 35,000 at 6 months. These values were significantly higher than FVB mice at all ages and significantly higher than ACKR2 mice at 4 and 6 months. Interestingly, ACR values of OVE-ACKR2 mice were significantly lower than OVE values at all ages. The difference between ACR levels of OVE and OVE-ACKR2 groups increased from about 2-fold at 2 months to about 15-fold at 4 months and 7-fold at 6 months.

#### 3.1.2. Reduced Renal Fibrosis and Inflammation in ACKR2 Mice

**Figure 3:**
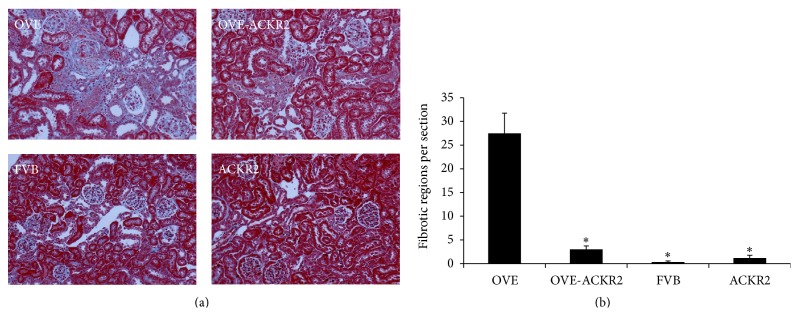
Renal fibrosis is reduced by knockout of the ACKR2 gene in diabetic OVE-ACKR2 mice. (a) Representative images of renal fibrosis illustrated by trichrome staining in a kidney section for each genotype. Original magnification 200x. (b) Scoring of renal fibrosis by blind counting of blue stained fibrotic regions in trichrome stained kidney sections. ^*∗*^ *p* < 0.02 versus OVE by one-way ANOVA. 6 sections from 3 mice per group were counted.

We evaluated the glomerular and tubular damage in OVE and OVE-ACKR2 mice at the age of 6 months as previously described [22–24]. Trichrome staining (Figure 3(a)) showed that fibrosis in OVE kidneys was much greater than in nondiabetic or OVE-ACKR2^−/−^ kidneys. Semiquantitative scoring of trichrome staining (Figure 3(b)) by an observer blind to genotype confirmed that deletion of the ACKR2 gene significantly reduced fibrosis in diabetic OVE-ACKR2 mice compared to OVE mice.

**Figure 4:**
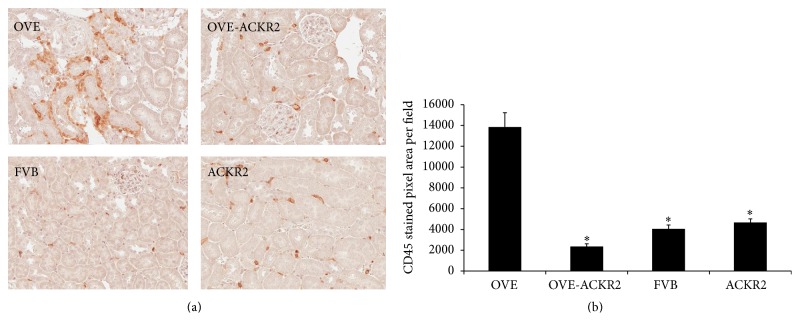
Knockout of the ACKR2 gene reduces leukocyte infiltration in diabetic mice. (a) Representative images of CD45 staining, original magnification 200x. (b) Quantitative analysis of leukocyte infiltration scored as CD45 positive pixel area per visual field. Twenty-four random fields from 3 mice per group were measured. *∗* indicates *p* < 0.05 versus OVE. Statistical comparisons were performed by one-way ANOVA.

Infiltration of leukocytes in kidney was determined by staining with anti-CD45 antibody (Figure 4). In nondiabetic FVB and ACKR2 mice, CD45 positive cells were sparsely distributed in the interstitial vessels and in the glomerular tuft. In OVE kidneys many more CD45 positive cells were observed, located mostly in the peritubular, interstitial space in a clustered distribution. Positive staining of CD45 cells was much less evident in kidneys of OVE-ACKR2 mice and appeared similar to staining in nondiabetic mice. Quantitation of CD45 positive pixel area confirmed significantly less leukocyte accumulation in the OVE-ACKR2 mice compared to the OVE mice (Figure 4(b)). Staining for CD3 to identify T cells demonstrated that CD3 positive cells were also more abundant in OVE kidneys than in any other genotype (data not shown).

**Figure 5:**
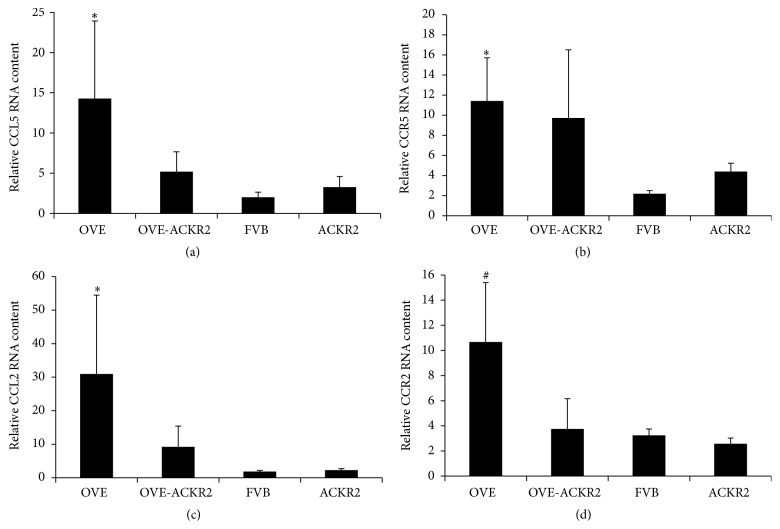
Kidney RNA levels of ACKR2 ligands CCL2 and CCL5 and their receptors. Values were determined by RT-PCR with Taqman probes using 18S as standard. Columns are mean + SE. *n* = 3 OVE, 4 OVE-ACKR2, 5 ACKR2, and 6 FVB. *∗* indicates *p* ≤ 0.05 versus FVB and # indicates a trend of *p* ≤ 0.08 versus FVB. All determined by one-way ANOVA.

Inflammatory chemokines CCL2 and CCL5 (ligands for ACKR2) are elevated in DN [6, 27, 28]. Quantitative RT-PCR for CCL2, CCL5, and their receptors was performed on RNA samples extracted from kidneys of all groups at 6 months of age (Figure 5). Levels of CCL2 and CCL5 mRNA significantly increased in OVE mice compared to FVB mice and OVE-ACKR2^−/−^ mice (Figure 5).

### 3.2. Microarray Analysis of Kidneys from OVE and OVE-ACKR2 Mice

The global changes in gene expression profiles were evaluated by microarray. To confirm the reliability of the microarray results correlation coefficients were calculated between RT-PCR and microarray results for CCL2, CCL5, CCR2, and CCR5 based on the 12 samples used in both assays. For all but CCR5 the correlation was at least 0.96 (*p* ≤ 0.000001) and for CCR5 the correlation coefficient was 0.6 (*p* ≤ 0.05).

Only 18 of 30,000 genes differed at the 0.05 level between the nondiabetic groups, FVB and ACKR2. Therefore, RNA expression of the OVE and OVE-ACKR2^−/−^ diabetic groups was compared to one nondiabetic group, FVB. Using a minimal criterion of 1.5-fold change in expression and a *p* value of 0.05 versus FVB, there were 715 genes in OVE, 181 in OVE-ACKR2, and 18 in ACKR2 samples that reached this criterion. Expression data was analyzed with Ingenuity Pathway Analysis (IPA) software.  Table 1 shows 40 IPA canonical pathways significantly affected by OVE diabetes arranged in 8 biological categories. Signaling pathways for hepatic fibrosis and leukocyte extravasation contained a large number of genes (26 and 27 genes, resp.) altered in expression in OVE samples. This is consistent with the extensive fibrosis and CD45 positive cell infiltration of OVE kidneys (Figures 3 and 4). OVE-ACKR2 kidneys, which showed minimal histological changes, had only 6 induced genes in the fibrosis pathway and 2 in the leukocyte extravasation pathway.

In OVE kidney, many protective pathways such as immune response and cytokine signaling were activated, as indicated by the high number of RNAs with significantly altered expression. The same pathways in OVE-ACKR2 contained only a few RNAs with altered expression. With few exceptions, most of the biological pathways in Table 1 contained at least 4 times as many significantly modified RNAs for OVE as they did for OVE-ACKR2. Also, only 5 of the 40 pathways significantly affected by OVE diabetes were significantly affected by OVE-ACKR2 diabetes. The conclusion that inflammation was reduced by deletion of ACKR2 was also evident at the individual RNA level: transcripts reduced in OVE-ACKR2 kidneys relative to OVE kidneys included RNAs indicative of complement activation (C7 and C1qc) and macrophage and T cell infiltration (Mpeg1, Cd68, and Itgam) and other cytokines (CCL8, CCL9, and CCL28) (Supplementary Tables 1 and 2, in Supplementary Material available online at http://dx.doi.org/10.1155/2016/5362506, show the 50 transcripts most reduced and increased in OVE-ACKR2 relative to OVE, resp.).

### 3.3. ACKR2 Protein Expression in Kidneys of Diabetic Patients

**Figure 6:**
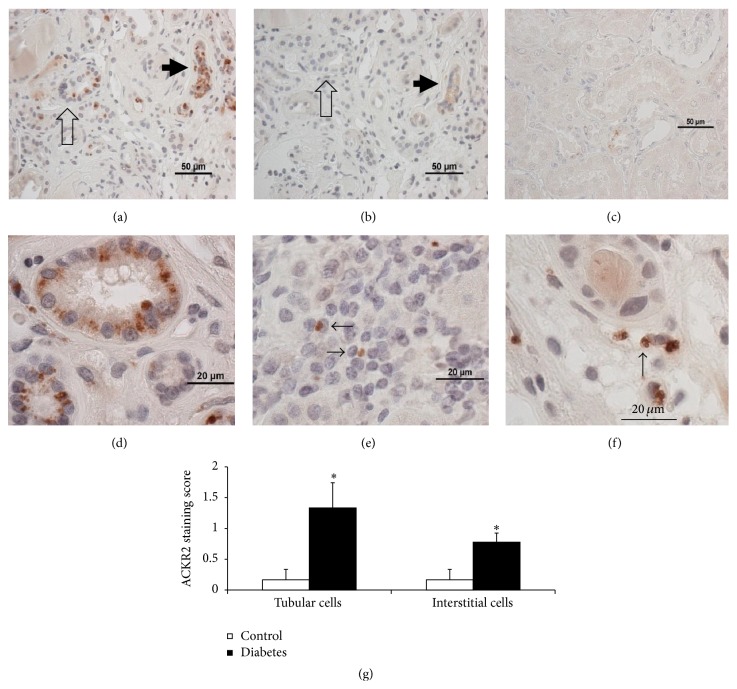
Increased ACKR2 protein in diabetic human kidney sections stained with rat monoclonal antibody to human ACKR2. (a) Positive ACKR2 staining in diabetic kidney. Strongest staining in tubules (arrows) especially in a collapsed (arrow) tubule. (b) Minimal staining is seen on a serial section without primary antibody. The arrows indicate the same 2 tubules in images (a) and (b). (c) Sparse ACKR2 staining in a nondiabetic section. (d) At higher magnification granule-like deposits of ACKR2 can be seen in cytoplasm of proximal tubular epithelial cells in diabetic kidney. In the interstitial space ACKR2 staining is also visible in diabetic kidney monocytes (e) and endothelial cells (f). (g) Semiquantitative scoring of ACKR2 staining by a scorer blind to sample identity. Scores for proximal tubule and interstitial cells are higher in diabetic than nondiabetic samples. ^*∗*^ *p* < 0.05 by *t*-test, *n* = 9 diabetic and 6 nondiabetic samples.

The effect of diabetes on kidney ACKR2 protein expression was evaluated in human DN and nondiabetic samples using a rat anti-human ACKR2 monoclonal antibody, previously evaluated on human samples [18, 29, 30]. A reliable antibody to mouse ACKR2 is not available. Positive but sporadic ACKR2 staining was visible in diabetic kidneys (Figures 6(a) and 6(d)–6(f)) in tubule epithelial cells and in the interstitium. Stained tubule epithelial cells were positively identified by the presence of a brush border by staining with* Lotus tetragonolobus* lectin [25]. ACKR2 staining was never seen in glomeruli. Positive cells in the interstitium appeared to be either mononuclear cells (lymphocytes or monocytes) or endothelial cells belonging to capillaries or lymphatics. Staining was more frequent and more intense in diabetic samples, which was confirmed by semiquantitative scoring of epithelial cells and interstitial cells (Figure 6(g)).

## 4. Discussion

This study demonstrates that the ACKR2 chemokine scavenger receptor has an unexpected important role in the development of diabetic kidney disease. Deletion of the ACKR2 gene in OVE diabetic mice produced a great reduction in albuminuria, accompanied by reduced severity of renal fibrosis, leucocyte infiltration, and inflammatory chemokine gene expression. In addition, ACKR2 protein content was elevated in several cell types in kidneys of DN patients.

Chemokines and cytokines regulate the inflammatory processes and contribute to progressive kidney damage in diabetes [31]. Chemokine scavenging has been proposed as a significant mechanism for controlling ongoing inflammation. This suggests that scavenger receptors like ACKR2 could limit DN progression by reducing kidney chemokine levels. Surprisingly little information is available for the ACKR2 chemokine scavenger receptor in the kidney, and only previous study indicated that the level of ACKR2 RNA in mouse kidney is low [15]. The current study also found low expression of ACKR2 RNA in normal kidney, approximately fiftyfold lower than in lung. We further observed a tendency for diabetes to increase ACKR2 RNA expression in OVE mouse kidney.

To determine if the ACKR2 RNA results indicate that diabetes alters ACKR2 protein immunohistochemistry studies were performed on human tissue since only an anti-human ACKR2 antibody has been validated [18, 29, 30]. Diabetic kidneys had significantly stronger ACKR2 staining in tubule and interstitial cells. Staining increased primarily in proximal tubule cells and in tubule cells that were too abnormal to distinguish as proximal or distal (Figure 6(a)). ACKR2 positive interstitial cells seen in diabetic samples appeared to be a mix of infiltrating monocytes and endothelial cells which could belong to blood or lymphatic vessels. The ACKR2 positive cell profile in kidney was not unusual. Positive stromal cells were expected, since ACKR2 staining in other organs has been reported for monocytes, lymphocytes, dendritic cells, and endothelial cells. Increased infiltration of inflammatory cells is common in diabetic kidneys [7, 8, 32]. Tubule cell staining for ACKR2 is unsurprising considering ACKR2 has been shown in parenchymal cells of several organs: ACKR2 antibodies stain epidermis in psoriatic skin [33], syncytiotrophoblast cells of placenta [18], and breast cancer cells [34]. The absence of ACKR2 staining in diabetic glomeruli indicates that direct actions of ACKR2 are limited to the tubular and interstitial portions of the diabetic kidney.

The primary finding of this study was that ACKR2 deletion dramatically reduced DN. The reduction of albuminuria in OVE-ACKR2 mice was significant at the earliest age tested, two months. As OVE mice aged DN progressed and the protection by ACKR2 KO became more striking. At 6 months ACKR2 deletion produced a greater reduction in diabetic albuminuria. In addition several markers demonstrated reduced inflammation in OVE-ACKR2 kidneys compared to OVE kidneys. Histologically this was indicated by decreased leukocyte infiltration and less fibrosis. Gene expression data demonstrated that absence of ACKR2 prevented activation of multiple molecular pathways involved in immune or inflammatory processes in kidneys of diabetic mice. The finding of such potent renal protection from diabetes by deletion of ACKR2 was contradictory to our expectation, which was that deletion of ACKR2 would exacerbate DN by increasing renal inflammation. This expectation was based on the damage inflammation produces in DN and the anti-inflammatory potency of ACKR2 as a scavenger of proinflammatory chemokines. In several studies manipulation of ACKR2 levels modified tissue inflammation in a manner that would be predicted based on anti-inflammatory potency of ACKR2 as a chemokine scavenger: this was shown in experimental models of colitis and psoriasis, where deletion of ACKR2 increased colon [21] or skin [17] inflammation, and in inflamed NOD mouse islets where transgenic overexpression of ACKR2 reduced local islet inflammation [35].

Mechanisms to explain protection from DN by deletion of ACKR2 are not obvious. Protection was not due to reduced OVE diabetes since hyperglycemia was equivalent in OVE and OVE-ACKR2 mice (Figure 1). In considering potential mechanisms of protection by ACKR2 KO it needs to be considered that this is not the first such report. Unexpected protection by deletion of ACKR2 has been reported to reduce pathology of several inflammatory diseases: ACKR2 deletion inhibits spinal cord inflammation and autoimmune encephalomyelitis [36], reduces susceptibility and symptoms of dextran sulfate-induced colitis [37], and reduces airway reactivity in allergen-induced airway disease [19]. In addition to these inflammatory disease models, KO of host ACKR2 can suppress transplant graft rejection [38, 39]. The unexpected but repeated finding of beneficial effects of ACKR2 deletion in multiple disease models indicates that our anti-inflammatory concept of ACKR2 was overly simplistic and the chemokine scavenging properties of ACKR2 may produce complex and not purely anti-inflammatory results. For example, deletion of ACKR2 releases chemokines that promote increased production of immunosuppressive monocytes [39] that reduce graft-versus-host disease. During chronic DN progression complex and changing interactions occur between the immune system and the kidney. At this time, the underlying molecular mechanisms involved in diabetic kidney disease are not clear. It is possible that kidney damage initiated by hyperglycemia is more efficiently cleared in ACKR2 mice. More rapid damage removal decreases the chances of developing chronic inflammation. Despite uncertainty about the mechanism, the strength of protection produced by elimination of ACKR2 indicates that it has a key role in the pathology which needs to be dissected at a finer level.

In* summary*, we found that deletion of the ACKR2 gene produced a dramatic reduction in albuminuria and renal inflammation in the OVE diabetic mouse without decreasing diabetes. In human samples diabetes increased the expression of ACKR2 protein in tubule cells, leukocytes, and endothelial cells.

## Supplementary Material

Supplement Tables 1 and 2 show the 50 transcripts most reduced (Table 1) and most increased (Table 2) in OVE-ACKR2 kidney compared to OVE kidney. Only OVE-ACKR2 transcripts that were significantly different from OVE p≤0.05 are included. Values are the average signal ±SEM from a sample size of 3.
Supplement Figure 1 images show that most ACKR2 positive cells in diabetic human kidneys are tubule epithelial cells. In the upper panel ACKR2 staining is red and the proximal tubule brush border is labeled green with Lotus tetragonolobus lectin, a marker for kidney tubule brush border. The lower panel is a DIC image showing that the central tubule has an almost continuous brush border adjacent to nearly continuous ACKR2 stained cells. In both images most ACKR2 staining is on tubule epithelial cells with a brush border.

## Figures and Tables

**Table 1:** Ingenuity pathways in kidney affected by OVE diabetes and/or OVE-ACKR2 diabetes.

Ingenuity canonical pathway	OVE versus FVB		OVE-ACKR2 versus FVB	
	*p* value	Ratio^*∗*^	*p* value	Ratio^*∗*^
*Diseases-specific pathways*				
Hepatic fibrosis	4.27*E* − 10	26/147	0.003	6/147
Atherosclerosis signaling	7.76*E* − 10	23/129	NS	2/129
Altered T cell and B cell signaling in rheumatoid arthritis	3.38*E* − 08	17/92	NS	1/92
Graft-versus-host disease signaling	3.71*E* − 06	10/50	NS	1/50
Glioma invasiveness signaling	6.31*E* − 06	12/60	NS	1/60
*Cellular immune response*				
Communication between innate and adaptive immune cells	3.89*E* − 10	18/109	NS	1/109
Dendritic cell maturation	2.39*E* − 08	23/185	NS	0
Altered T cell and B cell signaling in rheumatoid arthritis	3.38*E* − 08	17/92	NS	1/92
Pattern recognition receptors of bacteria and viruses	3.38*E* − 08	19/106	NS	2/106
Leukocyte extravasation signaling	6.61*E* − 08	27/199	NS	2/199
*Humoral immune response*				
Complement system	7.94*E* − 11	13/35	NS	1/35
B cell development	5.49*E* − 06	8/36	NS	0
NF-*κ*B signaling	7.94*E* − 05	19/175	NS	0
p38 MAPK signaling	0.00017	14/106	NS	0
Antigen presentation pathway	0.0002	7/40	NS	0
*Intracellular and second messenger signaling*				
p38 MAPK signaling	0.0002	14/106	NS	0
Role of NFAT in regulation of the immune response	0.002	16/198	NS	0
Nitrogen metabolism	0.0037	6/120	NS	1/120
Histidine metabolism	0.0044	7/112	0.00012	5/112
Arginine and proline metabolism	0.0141	8/176	0.00676	4/176
*Cellular stress and injury*				
Intrinsic prothrombin activation pathway	1.55*E* − 05	8/32	NS	1/32
Coagulation system	1.73*E* − 05	9/38	NS	0
Extrinsic prothrombin activation pathway	5.25*E* − 05	6/20	NS	0
p38 MAPK signaling	0.00017	14/106	NS	0
HMGB1 signaling	0.00245	11/100	NS	0
*Cytokine signaling*				
Dendritic cell maturation	2.39*E* − 08	23/185	NS	0
Acute phase response signaling	8.91*E* − 08	25/177	0.00813	6/177
TREM1 signaling	6.92*E* − 05	10/66	NS	0
IL-8 signaling	7.41*E* − 05	20/193	NS	2/193
NF-*κ*B signaling	7.94*E* − 05	19/175	NS	0
*Pathogen-influenced signaling*				
Dendritic cell maturation	2.39*E* − 08	23/185	NS	0
Pattern recognition receptors of bacteria and viruses	3.38*E* − 08	19/106	NS	2/106
Virus entry via endocytic pathways	0.00014	13/100	NS	2/100
Clathrin-mediated endocytosis signaling	0.00019	20/195	NS	2/195
Caveolar-mediated endocytosis signaling	0.0015	10/85	NS	1/85
*Nuclear receptor signaling*				
LXR/RXR activation	1.63*E* − 13	28/136	0.0002	7/136
TR/RXR activation	0.0017	11/96	NS	2/96
Aryl hydrocarbon receptor signaling	0.0028	14/159	NS	6/159
Nitrogen metabolism	0.0037	6/120	NS	1/120
LPS/IL-1 mediated inhibition of RXR function	0.0039	18/235	NS	9/235

^*∗*^Ratio: RNAs altered versus FVB divided by the number of genes in the pathway. NS, not significant.
