# Supplementary material for: Renal Protection by Genetic Deletion of the Atypical Chemokine Receptor ACKR2 in Diabetic OVE Mice
Source: J Diabetes Res. 2015 Dec 20;2016:5362506. doi: 10.1155/2016/5362506 (PMC4699014; doi:10.1155/2016/5362506)
Supplement: Supplementary file 1 — Supplement Tables 1 and 2 show the 50 transcripts most reduced (Table 1) and most increased (Table 2) in OVE-ACKR2 kidney compared to OVE kidney. Only OVE-ACKR2 transcripts that were significantly different from OVE p≤0.05 are included. Values are the average signal ±SEM from a sample size of 3. Supplement Figure 1 images show that most ACKR2 positive cells in diabetic human kidneys are tubule epithelial cells. In the upper panel ACKR2 staining is red and the proximal tubule brush border is labeled green with Lotus tetragonolobus lectin, a marker for kidney tubule brush border. The lower panel is a DIC image showing that the central tubule has an almost continuous brush border adjacent to nearly continuous ACKR2 stained cells. In both images most ACKR2 staining is on tubule epithelial cells with a brush border. [file 5362506.f1.pdf]

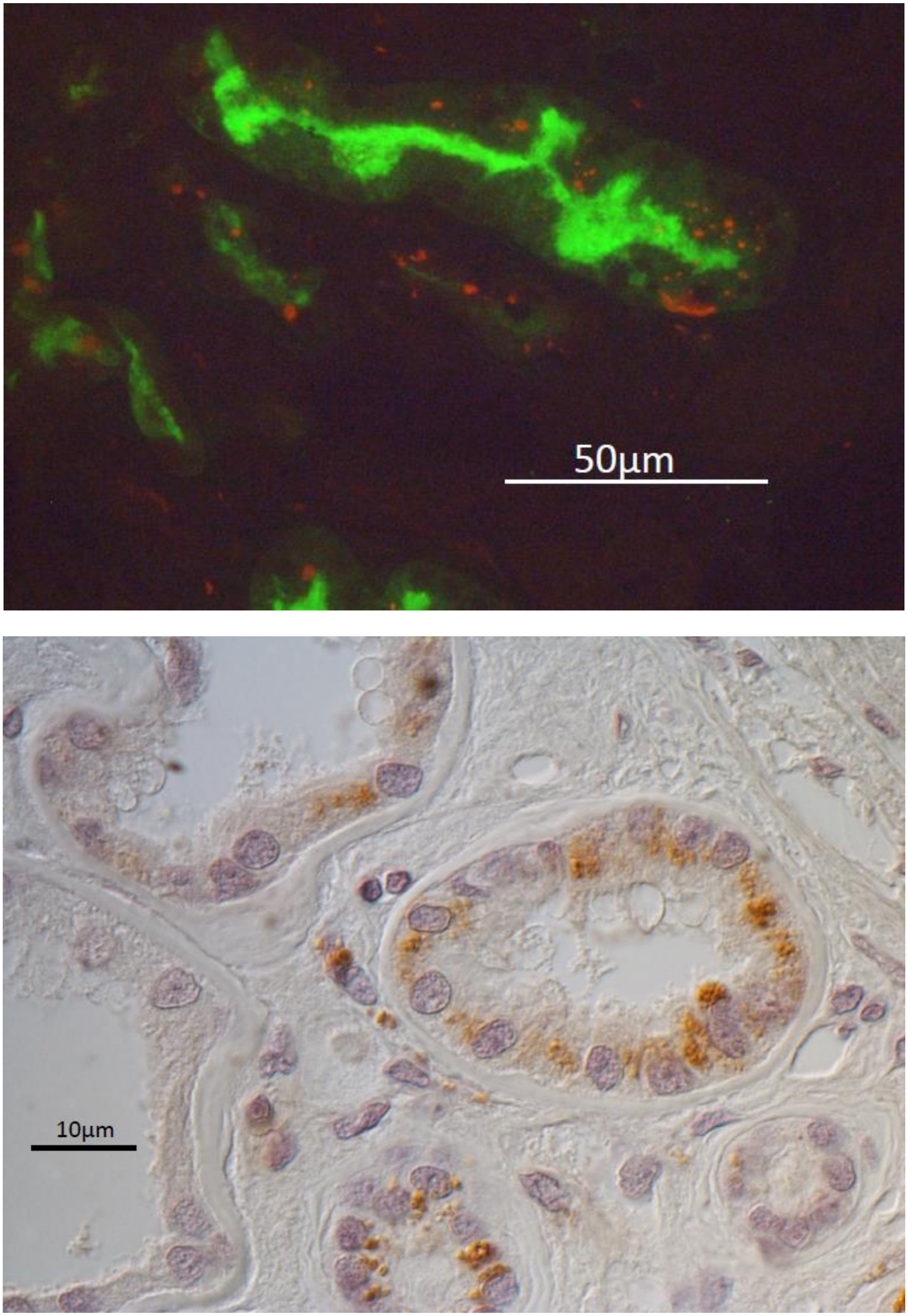

Supplement Figure 1.Most ACKR2 positive cells in diabetic human kidneys are tubule epithelial cells: The upper panel shows ACKR2 staining in red (Cy3) and green labeling of the proximal tubule brush border with FITC labeled *Lotus tetragonolobus* lectin, a specific marker in kidney for tubule epithelial cell brush border [25]. The lower panel is a differential interference contrast (DIC) image clearly showing that the central tubule has an almost continuous brush border adjacent to nearly continuous ACKR2 stained cells. In both images the predominance of ACKR2 staining on cells with a brush border indicates that most ACKR2 stained cells are tubule epithelial cells.

Supplement Table 1: 50 transcripts most reduced in OVE-ACKR2 kidney compared to OVE kidney among all OVE-ACKR2 transcripts significantly different from OVE by ANOVA P≤0.05. Values are the average signal ±SEM, n=3 .

RATIO OVE / OVE-ACKR2	p-value OVE vs OVE-ACKR2	gene_assignment	Gene Symbol	OVE signal	OVE-ACKR2 signal	FVB signal	ACKR2 signal
6.5	0.033	immunoglobulin kappa chain variable 1	Igk-V1	693±412	106±19	200±18	106±19
5.7	0.003	lipocalin 2	Lcn2	1663±772	291±189	108±10	291±189
5.3	0.043	immunoglobulin lambda chain, variable 2	Igl-V2	296±175	56±6	76±5	56±6
4.8	0.000	chemokine (C-C motif) receptor 2	Ccr2	179±49	37±2	59±12	37±2
4.3	0.017	lysozyme 1	Lyz1	231±117	54±6	40±.4	54±6
4.3	0.013	immunoglobulin kappa chain variable 28	Igk-V28	673±318	157±16	292±21	157±16
4.2	0.002	lysozyme 2	Lyz2	3352±1161	794±97	443±66	794±97
4.2	0.022	immunoglobulin joining chain	Igj	784±360	189±12	355±14	189±12
4.1	0.006	serine peptidase inhibitor, clade A 3N	Serpina3n	978±435	240±65	175±19	240±65
3.8	0.038	immunoglobulin heavy variable V1-72	Ighv1-72	711±336	186±38	289±7	186±38
3.8	0.013	complement component 7	C7	1092±436	287±84	247±15	287±84
3.8	0.018	membrane-spanning 4-domains, A, 6D	Ms4a6d	147±64	39±5	32±3	39±5
3.6	0.030	ribonuclease P RNA-like 1	Rprl1	262±118	73±10	123±11	73±10
3.5	0.014	keratin 20	Krt20	137±81	39±9	31±3	39±9
3.5	0.033	chemokine (C-C motif) ligand 28	Ccl28	805±337	232±51	156±33	232±51
3.5	0.011	toll-like receptor 8	Tlr8	88±37	25±3	23±1	25±3
3.3	0.001	CD44 antigen	Cd44	290±111	89±9	77±4	89±9
3.2	0.008	membrane-spanning 4-domains, A, 6C	Ms4a6c	522±189	164±14	127±10	164±14
3.1	0.005	hepatitis A virus cellular receptor 1	Havcr1	901±268	289±105	180±24	289±105
3.1	0.025	eosinophil-associated, ribonuclease A 10	Ear10	293±170	94±10	58±4	94±10
3.1	0.002	interleukin 1 family, member 6	Il1f6	98±37	32±4	28±2	32±4
3.0	0.005	EGF-like, mucin-like, hormone receptor-like	Emr1	291±93	98±4	99±24	98±4
3.0	0.001	matrix metallopeptidase 12	Mmp12	197±74	67±3	68±4	67±3
2.9	0.005	tissue inhibitor of metalloproteinase 1	Timp1	215±89	74±16	52±2	74±16
2.9	0.018	complement component 1, q C chain	C1qc	492±193	170±34	157±25	170±34
2.9	0.009	chemokine (C-C motif) ligand 2	Ccl2	254±121	88±11	59±4	88±11
2.9	0.001	chemokine (C-C motif) ligand 9	Ccl9	221±69	77±10	65±4	77±10
2.8	0.037	CD74 antigen	Cd74	3089±1145	1101±30	930±224	1101±30
2.8	0.016	CD53 antigen	Cd53	447±176	160±7	123±3	160±7
2.8	0.022	eosinophil-associated, ribonuclease A 1	Ear1	231±125	83±7	57±3	83±7
2.7	0.002	vascular cell adhesion molecule 1	Vcam1	650±206	238±32	241±10	238±32
2.7	0.023	CD209a antigen	Cd209a	212±101	79±14	84±13	79±14
2.6	0.012	interleukin 7 receptor	Il7r	102±39	39±2	37±1	39±2
2.6	0.029	chemokine (C-C motif) ligand 8	Ccl8	75±35	29±1	24±1	29±1
2.6	0.014	fibrinogen gamma chain	Fgg	636±154	246±96	193±25	246±96
2.6	0.008	macrophage expressed gene 1	Mpeg1	370±120	144±8	125±13	144±8
2.6	0.010	cathepsin S	Ctss	373±120	145±11	127±12	145±11
2.5	0.007	FYN binding protein	Fyb	289±88	114±5	105±11	114±5
2.5	0.025	toll-like receptor 7	Tlr7	240±84	96±17	101±17	96±17
2.5	0.002	serine peptidase inhibitor, clade Aalpha-1	Serpina10	217±31	87±22	78±14	87±22
2.5	0.001	mannose receptor, C type 1	Mrc1	376±95	153±6	143±6	153±6
2.4	0.004	complement component 3a receptor 1	C3ar1	633±163	260±31	216±15	260±31
2.4	0.015	aldehyde dehydrogenase family 1, A2	Aldh1a2	506±212	211±14	227±36	211±14
2.4	0.022	lymphocyte antigen 86	Ly86	601±201	253±17	223±31	253±17
2.4	0.008	chemokine (C-C motif) ligand 6	Ccl6	299±88	126±23	100±1	126±23
2.4	0.004	CD68 antigen	Cd68	425±135	179±21	123±6	179±21
2.3	0.021	toll-like receptor 13	Tlr13	122±44	52±5	41±2	52±5
2.3	0.001	chloride channel calcium activated 2	Clca2	311±73	133±10	82±3	133±10
2.3	0.008	integrin alpha M	Itgam	177±53	76±4	63±2	76±4

Supplement Table 2: 50 transcripts most increased in OVE-ACKR2 kidney compared to OVE kidney among all OVE-ACKR2 transcripts significantly different from OVE by ANOVA P≤0.05. Values are the average signal ±SEM, n=3 .

RATIO OVE / OVE-ACKR2	p-value OVE vs OVE-ACKR2	gene_assignment	Gene Symbol	OVE signal	OVE-ACKR2 signal	FVB signal	ACKR2 signal
0.23	0.007	serine peptidase inhibitor, clade A 6	Serpina6	612±480	2661±700	144±43	277±25
0.34	0.005	interferon induced transmembrane protein 7	Ifitm7	135±24	392±117	189±49	367±67
0.49	0.020	deoxyribonuclease I	Dnase1	661±84	1348±367	788±111	1013±246
0.53	0.005	4-hydroxyphenylpyruvic acid dioxygenase	Hpd	693±47	1316±222	1374±294	1531±145
0.53	0.027	cytochrome P450, family 4a, polypeptide 12a	Cyp4a12a	1242±279	2343±138	569±184	433±77
0.54	0.035	insulin-like growth factor binding protein 1	Igfbp1	147±54	274±42	195±31	168±39
0.57	0.012	sal-like 2 (Drosophila)	Sall2	34±4	60±8	49±11	39±3
0.58	0.047	small nucleolar RNA, C/D box 14C	Snord14c	1372±363	2355±502	2305±332	1550±260
0.58	0.004	B-cell leukemia/lymphoma 2	Bcl2	54±9	93±13	56±3	77±7
0.60	0.037	olfactory receptor 1392	Olfr1392	78±12	130±33	80±7	96±5
0.60	0.021	nephrocan	Nepn	91±24	151±28	208±20	286±7
0.62	0.001	solute carrier family 6	Slc6a20a	1154±167	1873±62	998±15	1485±120
0.63	0.012	solute carrier family 8 sodium/calcium	Slc8a1	918±86	1466±169	1158±134	1647±263
0.63	0.000	Cbp/p300-interacting transactivator	Cited1	184±19	293±19	237±9	255±9
0.63	0.002	olfactory receptor 1393	Olfr1393	140±7	222±22	144±20	160±1
0.64	0.000	kallikrein related-peptidase 14	Klk14	28±3	44±2	40±2	50±3
0.64	0.000	parvalbumin	Pvalb	289±23	451±26	517±25	603±35
0.65	0.033	aldo-keto reductase family 1, member D1	Akr1d1	227±37	350±53	239±32	224±29
0.65	0.000	DEAD (Asp-Glu-Ala-Asp) box polypeptide 43	Ddx43	38±2	59±6	36±2	58±
0.65	0.027	aldo-keto reductase family 1, member B7	Akr1b7	52±3	80±18	62±3	71±6
0.65	0.020	aquaporin 8	Aqp8	56±4	87±18	51±5	60±5
0.66	0.004	carnosine dipeptidase 1	Cndp1	558±78	848±41	969±88	917±44
0.67	0.029	secreted phosphoprotein 2	Spp2	1310±338	1955±125	1642±152	1953±60
0.67	0.031	olfactory receptor 459	Olfr459	19±1	29±2	26±6	24±2
0.68	0.003	olfactory receptor 1022	Olfr1022	12±1	17±2	15±1	16±1
0.68	0.048	MAS-related GPR, member H	Mrgprh	45±8	67±10	56±3	63±10
0.68	0.011	zinc finger protein 616	Zfp616	9±	14±2	11±1	11±1
0.68	0.035	procollagen C-endopeptidase enhancer 2	Pcolce2	233±24	342±43	313±54	285±33
0.68	0.048	cDNA sequence BC037703	BC037703	98±15	144±24	68±9	74±3
0.68	0.003	guanylate cyclase 2f	Gucy2f	28±2	40±4	40±2	41±2
0.69	0.016	transducin-like enhancer of split 2	Tle2	190±25	276±9	293±47	243±21
0.69	0.010	phosphatidylinositol glycan anchor synthesis Z	Pigz	319±33	463±61	200±16	261±17
0.69	0.024	elongation factor RNA polymerase II-like 3	Ell3	185±8	269±36	166±9	210±36
0.69	0.009	vomeronasal 1 receptor 27	Vmn1r27	25±3	37±2	39±4	31±3
0.69	0.015	transient receptor potential cation channel M	Trpm6	325±21	470±60	481±54	532±51
0.69	0.043	stimulated by retinoic acid gene 6	Stra6	352±25	509±87	263±36	343±36
0.69	0.009	Rhesus blood group-associated B glycoprotein	Rhbg	556±24	802±23	818±89	966±125
0.69	0.002	tRNA-yW synthesizing protein 3 homolog	Tyw3	359±39	517±34	417±18	518±26
0.70	0.047	betaine-homocysteine methyltransferase	Bhmt	833±35	1188±151	1089±191	1019±130
0.70	0.011	solute carrier family 13 sodium/sulfate	Slc13a4	132±12	187±13	176±6	166±24
0.70	0.044	renin 1 structural	Ren1	764±88	1084±220	1944±67	2724±95
0.71	0.005	inosine 5'-phosphate dehydrogenase 2	Impdh2	43±3	61±8	38±1	46±3
0.71	0.027	aminoacylase 1	Acy1	2527±395	3575±257	2807±351	2692±274
0.71	0.014	testis expressed gene 19.1	Tex19.1	30±3	43±6	48±4	53±1
0.71	0.015	glycerophosphodiester phosphodiesterase 3	Gdpd3	1280±215	1808±112	1511±119	1860±134
0.71	0.022	solute carrier family 34 sodium phosphate 3	Slc34a3	1796±321	2522±167	2115±210	2461±145
0.71	0.012	phosphatidylinositol 3-kinase, C2	Pik3c2g	789±117	1107±49	1287±104	1356±84
0.71	0.015	Fc receptor, IgA, IgM, high affinity	Fcamr	76±3	106±12	182±21	197±17
0.71	0.000	defensin beta 4	Defb4	12±1	16±1	14±1	15±1
0.72	0.002	proteasome, beta type 7 pseudogene	Gm6787	46±4	64±4	59±3	63±2
